# Sequential inflammatory processes define human progression from *M*. *tuberculosis * infection to tuberculosis disease

**DOI:** 10.1371/journal.ppat.1006687

**Published:** 2017-11-16

**Authors:** Thomas J. Scriba, Adam Penn-Nicholson, Smitha Shankar, Tom Hraha, Ethan G. Thompson, David Sterling, Elisa Nemes, Fatoumatta Darboe, Sara Suliman, Lynn M. Amon, Hassan Mahomed, Mzwandile Erasmus, Wendy Whatney, John L. Johnson, W. Henry Boom, Mark Hatherill, Joe Valvo, Mary Ann De Groote, Urs A. Ochsner, Alan Aderem, Willem A. Hanekom, Daniel E. Zak, other members of the ACS cohort study team

**Affiliations:** 1 South African Tuberculosis Vaccine Initiative, Institute of Infectious Disease and Molecular Medicine & Division of Immunology, Department of Pathology, University of Cape Town, Cape Town, South Africa; 2 The Center for Infectious Disease Research, Seattle, WA, United States of America; 3 Somalogic Inc, Boulder, CO, United States of America; 4 Now at Metro District Health Services, Western Cape Government: Health and Division of Public Health and Health Systems, Department of Global Health, Faculty of Health Sciences and Medicine, Stellenbosch University, Cape Town, South Africa; 5 Tuberculosis Research Unit, Department of Medicine, Case Western Reserve University and University Hospitals Case Cleveland Medical Center, Cleveland, OH, United States of America; University of Massachusetts Medical School, UNITED STATES

## Abstract

Our understanding of mechanisms underlying progression from *Mycobacterium tuberculosis* infection to pulmonary tuberculosis disease in humans remains limited. To define such mechanisms, we followed *M*. *tuberculosis*-infected adolescents longitudinally. Blood samples from forty-four adolescents who ultimately developed tuberculosis disease (“progressors”) were compared with those from 106 matched controls, who remained healthy during two years of follow up. We performed longitudinal whole blood transcriptomic analyses by RNA sequencing and plasma proteome analyses using multiplexed slow off-rate modified DNA aptamers. Tuberculosis progression was associated with sequential modulation of immunological processes. Type I/II interferon signalling and complement cascade were elevated 18 months before tuberculosis disease diagnosis, while changes in myeloid inflammation, lymphoid, monocyte and neutrophil gene modules occurred more proximally to tuberculosis disease. Analysis of gene expression in purified T cells also revealed early suppression of Th17 responses in progressors, relative to *M*. *tuberculosis*-infected controls. This was confirmed in an independent adult cohort who received BCG re-vaccination; transcript expression of interferon response genes in blood prior to BCG administration was associated with suppression of IL-17 expression by BCG-specific CD4 T cells 3 weeks post-vaccination. Our findings provide a timeline to the different immunological stages of disease progression which comprise sequential inflammatory dynamics and immune alterations that precede disease manifestations and diagnosis of tuberculosis disease. These findings have important implications for developing diagnostics, vaccination and host-directed therapies for tuberculosis.

**Trial registration:**

Clincialtrials.gov, NCT01119521

## Author summary

To define biological mechanisms that underlie progression of *Mycobacterium tuberculosis* infection to active tuberculosis, we followed *M*. *tuberculosis*-infected adolescents longitudinally. Those who ultimately developed tuberculosis disease (“progressors”) were compared with matched controls, who remained healthy. Whole blood transcriptomic and plasma proteome analyses showed sequential modulation of immunological processes. Type I/II interferon signalling and complement cascade were elevated 18 months before tuberculosis diagnosis, while changes in myeloid inflammation, lymphoid, monocyte and neutrophil responses occurred more proximally to tuberculosis disease. Analysis of gene expression in purified T cells revealed early suppression of Th17 responses in progressors. This was confirmed in an adult BCG re-vaccination cohort, where expression of interferon response genes in blood was associated with suppression of IL-17 expression by BCG-specific CD4 T cells. We concluded that sequential inflammatory dynamics and immune alteration precede tuberculosis disease manifestations, with important implications for developing diagnostics, vaccines and host-directed therapies for tuberculosis.

## Introduction

Almost a quarter of the global population is infected with *Mycobacterium tuberculosis* (*M*.*tb*) [1], placing millions at risk for tuberculosis (TB) disease. Five to 15% of infected individuals progress to active TB disease within their lifetimes. A spectrum of clinical disease severity may occur, associated with poorly regulated immune responses, including infection site and systemic inflammation and in some cases excessive inflammation [2,3]. TB disease-driven inflammation in the lung is characterized by presence of activated neutrophils, macrophages and lymphoid tissues in infected foci, as well as the presence of soluble inflammatory mediators at sites of disease [4–7]. Disease-driven systemic inflammation manifests with high levels of cytokines, chemokines, acute phase proteins, and other inflammatory mediators detectable in peripheral blood [8–10]. Peripheral blood transcriptomic profiling of patients with active TB disease demonstrated elevation of inflammatory gene expression pathways, including interferon (IFN) stimulated genes (ISG), myeloid inflammatory genes, and FC receptor/complement pathway genes [11–14].

There is limited knowledge about blood transcriptomic changes prior to development of clinical TB disease. We recently identified and validated a whole blood transcriptional signature of risk of TB disease, detectable long before disease manifests [15]. This 16-gene signature likely represents a small fraction of the host immunological changes that characterize progression to active TB disease [11–14]. Here, we proposed that a more extensive interrogation of immunological changes prior to clinical disease manifestations would enhance understanding of TB disease progression. We hypothesized that the spectrum of outcomes of *M*.*tb* infection–from asymptomatic quiescent infection, to subclinical disease (detectable only by special investigation), to symptomatic clinical TB disease [16]–is reflected by specific peripheral blood immunological and inflammatory profiles. We therefore applied a new in-depth transcriptomic, proteomic, and cellular analysis of blood collected from the adolescent cohort that was used to discover the above-mentioned whole blood signature of risk for TB disease. We also examined blood from an independent cohort of adults revaccinated with Bacille Calmette-Guérin (BCG) to validate functional adaptive immune response profiles that we found were associated with the inflammatory profiles observed during disease progression.

## Results

### Study participant cohorts and blood collection

#### Blood from two independent cohorts was analysed

**Fig 1:**
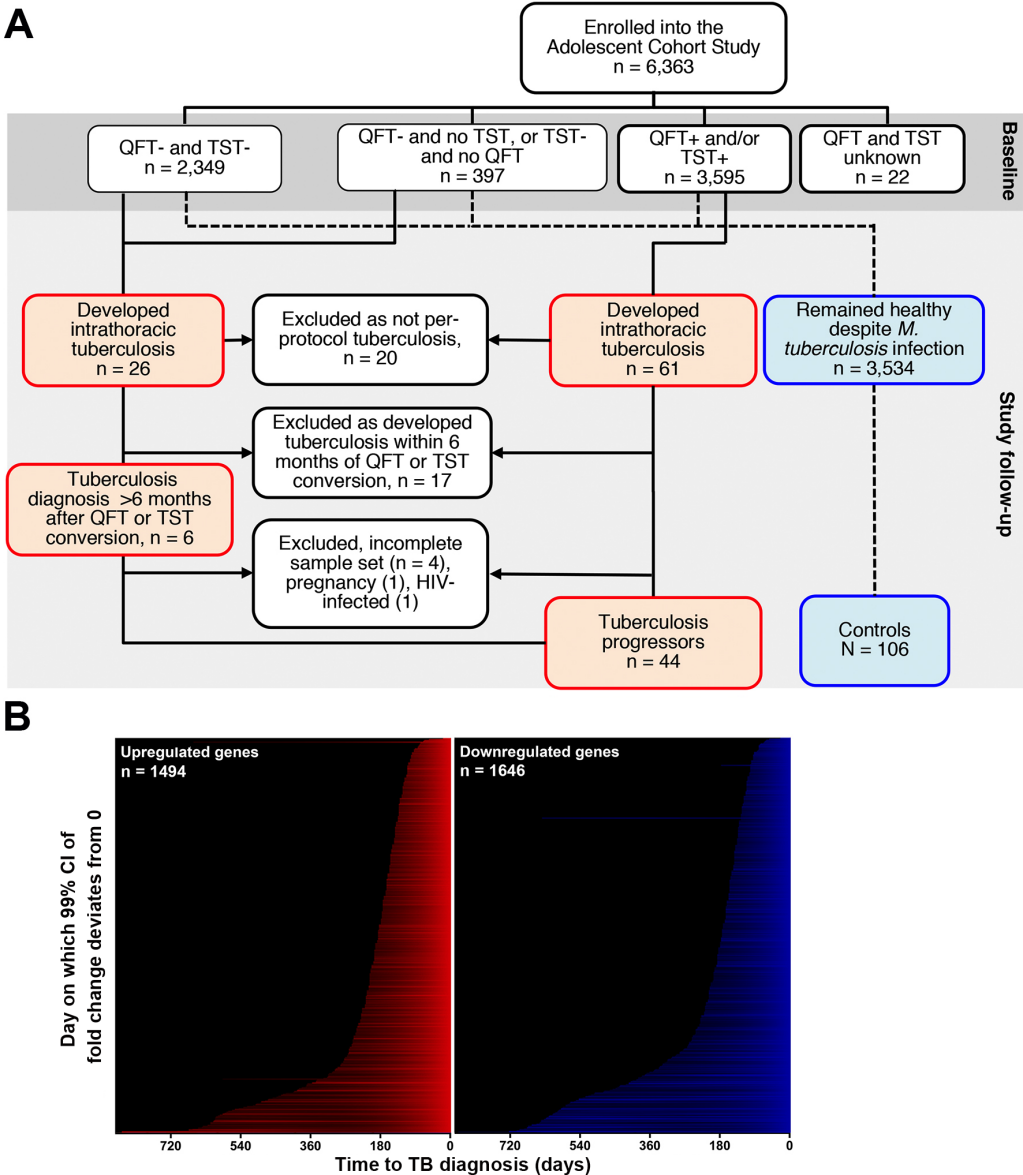
Kinetics of whole blood transcriptional responses during progression from infection to TB disease. (A) Consort diagram showing participant selection of the progressor and control substudy from the Adolescent Cohort Study (ACS). A total of 6,363 adolescents (12–18 years of age) were enrolled into the ACS. Participants were stratified according to their baseline *M*.*tb-*infection status according to either QFT-positive (≥0.35 IU/ml) and/or TST induration ≥ 10mm. Individuals with unknown QFT and TST test results were excluded. Participants with baseline *M*.*tb*-infection or who were QFT-negative and TST-negative at baseline but converted their tests at a later time point were eligible for inclusion as progressors or controls. Progressors developed intrathoracic TB disease, defined as TB diagnosis by at least two consecutive sputum smear positive tests, or at least one microbiologically confirmed culture positive test, at least 6 months after detection of *M*.*tb-*infection. Progressors were matched to healthy *M*.*tb-*infected “controls” based on age, gender, ethnicity, school, and any prior history of TB disease at a ~1:2 ratio. (B) Genes found to be significantly up (red) or down (blue) regulated in progressors relative to controls, ranked according to the time to TB disease at which expression in progressors (n = 38) is significantly different to controls (n = 104) (see **S1 Fig**). The full list of significantly regulated genes is in **S2 Table**.

First, 6,363 healthy adolescents, aged 12–18 years and enrolled into the Adolescent Cohort Study (ACS) [15], were followed for 24 months or more. Blood was collected at 6-monthly intervals. Among those with evidence of *M*.*tb* infection (QuantiFERON TB GOLD In-Tube Assay (QFT) and/or tuberculin skin test (TST) positive), 44 ultimately developed microbiologically confirmed TB disease >6 months after study enrolment; they were designated “progressors” (**Fig 1A, Table 1 and S1 Table**). Among progressors, the time from blood collection to diagnosis of active TB (“time to diagnosis”) ranged from 1 to 894 days. Realigning blood collection to “time to diagnosis” allowed serial assessment of transition from infection to TB disease (**S1 Table**). Controls (n = 106), who remained asymptomatic, were matched to progressors by age, gender, ethnicity, school and prior history of TB (**Table 1 and S1 Table**).

**Table 1:** Demographic details of TB progressor and control participants. Participants and sample numbers available for each analysis are also shown.

	Progressors (n = 44)	Controls (n = 106)	Total (n = 150)
Demographics			
Ethnicity, n (%)			
Coloured (Cape mixed ancestry)	40 (90.9%)	97 (91.5%)	137 (91.3%)
Black African	4 (9.1%)	9 (8.5%)	13 (8.7%)
Female, n (%)	32 (73%)	69 (65%)	101 (67.3%)
Mean age at baseline, years (range)	15.5 (12–18)	15.6 (13–18)	15.6 (12–18)
Prior episode of TB, n (%)	5 (11.4%)	16 (15.1%)	21 (14%)
RNA-Seq Transcriptomic Analysis			
Participants, n	38	104	142
Samples, n	76	243	319
Proteomic Analysis			
Participants, n	36	104	70
Samples, n	82	290	372
Flow Cytometry Analysis			
Participants, n	33	71	104
Samples, n	101	133	234

Second, 82 healthy and strongly TST positive adults were revaccinated with BCG [17] and blood was collected before and three weeks after BCG revaccination for analysis.

### Host transcriptional kinetics of TB progression

**Fig 2:**
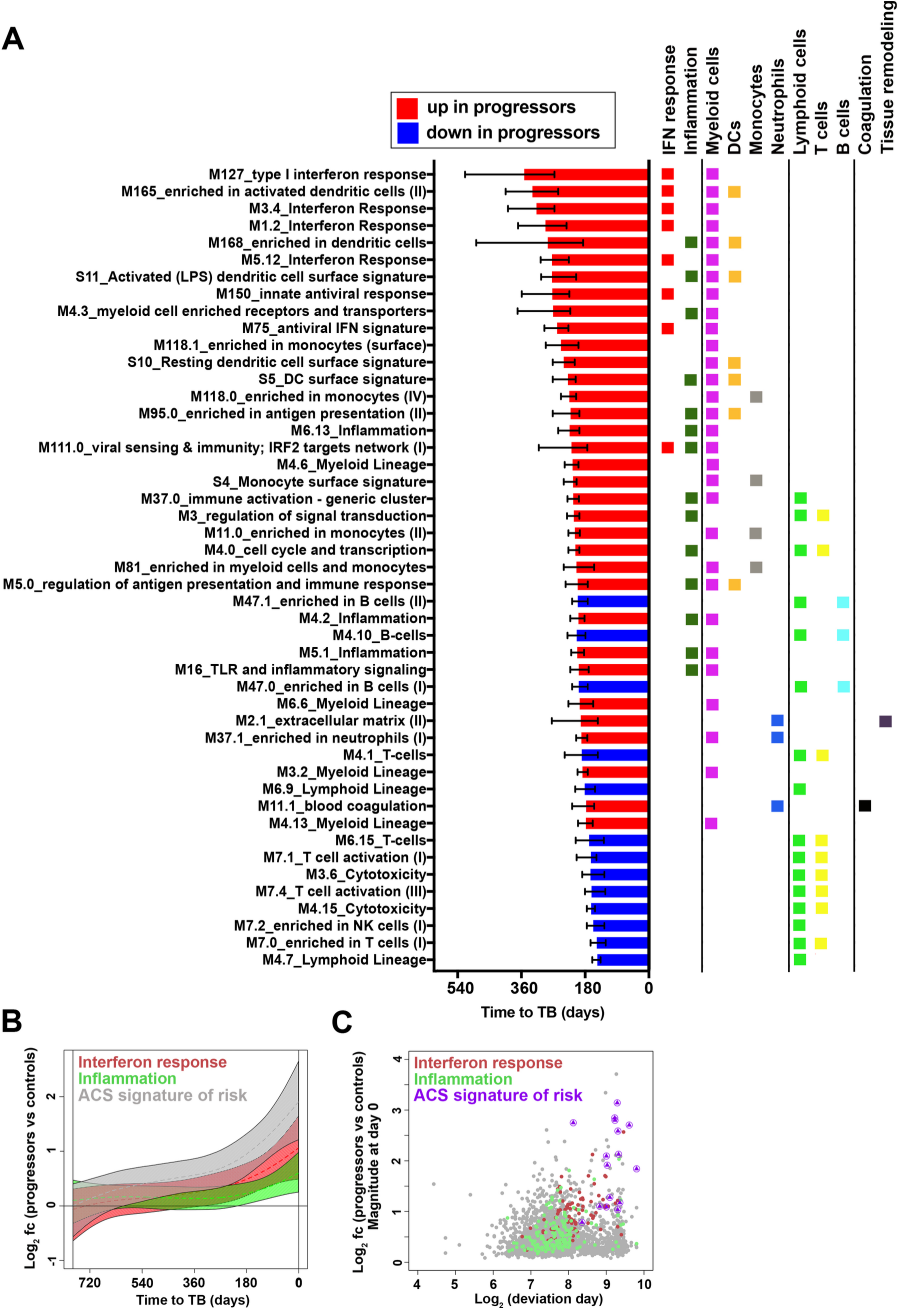
Sequential changes in distinct transcriptional modules during progression from *M*.*tb* infection to TB disease. **(A**) Gene modules, pre-defined by Chaussabel and BTM, found to be significantly enriched in progressors, compared with controls, and ranked in descending order according to median deviation time points (indicated by bars) of genes differentially expressed between progressors and controls. Data from 38 progressors and 104 controls were included in the analysis. Error bars denote IQR of median deviation time points of differentially expressed genes within each module. Assignment of each module to known immunological responses or processes or cellular subsets, according to differentially expressed genes, is indicated by the colored squares. The full list of significantly enriched modules is in **S4 Table**. (**B**) Kinetics of type I/II interferon response or inflammation transcriptional gene modules, as well as the 16 genes in the ACS signature of risk of TB. For interferon responses we included genes with significant kinetic response from modules: M127_type I interferon response, M5.12_Interferon Response, M3.4_Interferon Response and M1.2_Interferon Response. For inflammation we included genes with significant kinetic response from modules: M6.13_Inflammation, M4.2_Inflammation, M5.1_Inflammation, M16_TLR and inflammatory signaling, M33_inflammatory response and M53_inflammasome receptors and signaling. Module kinetics during progression were modeled as non-linear splines and 99% CI (shaded areas) were computed by performing 2000 spline fitting iterations after bootstrap resampling from the full dataset. (**C**) Scatter plot showing fold change (log_2_ FC) plotted versus the time point at which the 99% CI deviates from a log_2_ fold change of 0 (log_2_ days before TB diagnosis) for genes in the IFN response and inflammation modules and the 16 genes in the ACS signature of risk of TB.

Whole blood RNA isolated from PAXgene tubes collected from ACS progressors and controls was analyzed by RNA Sequencing (RNA-Seq). Nonlinear kinetic analysis of gene expression differences between the two groups was assessed over time, beginning 2 years before TB diagnosis of progressors. During progression to TB 1,494 genes were upregulated and 1,646 genes were downregulated in progressors, compared with controls. A wide range of kinetic changes in longitudinal expression of different genes was discernible (**Fig 1B** and **S2 Table**). Transcriptional module enrichment analysis revealed that a hierarchy of biological processes drove the development of progression-associated changes in gene expression (**Fig 2A** and **S3 Table**). Up-regulation of IFN response modules (containing ISGs such as STAT1, STAT2, IFITs, GBPs, MX1, OAS1 and IRF1), preceded up-regulation of myeloid inflammation and monocyte modules (containing MyD88, ICAM1, IL-6 amongst many others) (**Fig 2A and 2B**, **S1A Fig**). These changes preceded down-regulation of modules associated with specific lymphocyte cell populations (**Fig 2A, S3 Table**). Further, differential expression of the 16 genes that comprise the previously described whole blood signature of risk for TB disease [15] preceded all other modules [18,19], including the IFN response modules themselves, and their induction magnitude was among the highest detected (**Fig 2C**). This result suggests that genes contained in the previously-described whole blood signature of risk comprise a novel subset of ISGs that were robustly differentially expressed at the earliest stages of TB progression.

Since monocytes were strongly implicated in the module enrichment analysis above, suggesting either changes in peripheral monocyte numbers or changes in monocyte gene expression, we determined whether genes in the signature of risk were up-regulated in isolated monocytes from progressors compared to controls. Expression of FCGR1A/B and SERPING1, the inflammasome adaptor ASC/PYCARD and NCF1 (p47phox), which mediates inflammatory signaling in TB [20], were increased in monocytes from progressors who expressed the whole blood signature of risk for TB, compared to controls who did not express the signature of risk (**S8 Table, S2A Fig**). These results indicate that expression of the signature of risk of TB in whole blood may, in part, derive from enhanced expression of these transcripts in the monocyte population, suggesting that systemic monocyte activation, in addition to increased monocyte abundance (see below), may comprise a hallmark of TB progression that precedes the neutrophil activation associated with disease [11].

### Soluble proteomic biomarkers of TB progression

We complemented blood transcriptional analysis with proteomic analysis of plasma collected longitudinally at the same time points prior to disease manifestation in progressors from the adolescent cohort. Relative concentrations of >3,000 proteins were quantified with multiplexed slow off-rate modified DNA aptamers (SOMAmer reagents) [21,22]. During progression to TB, levels of 179 plasma proteins increased while 251 decreased, compared with controls (**S1B Fig, S4 Table**).

**Fig 3:**
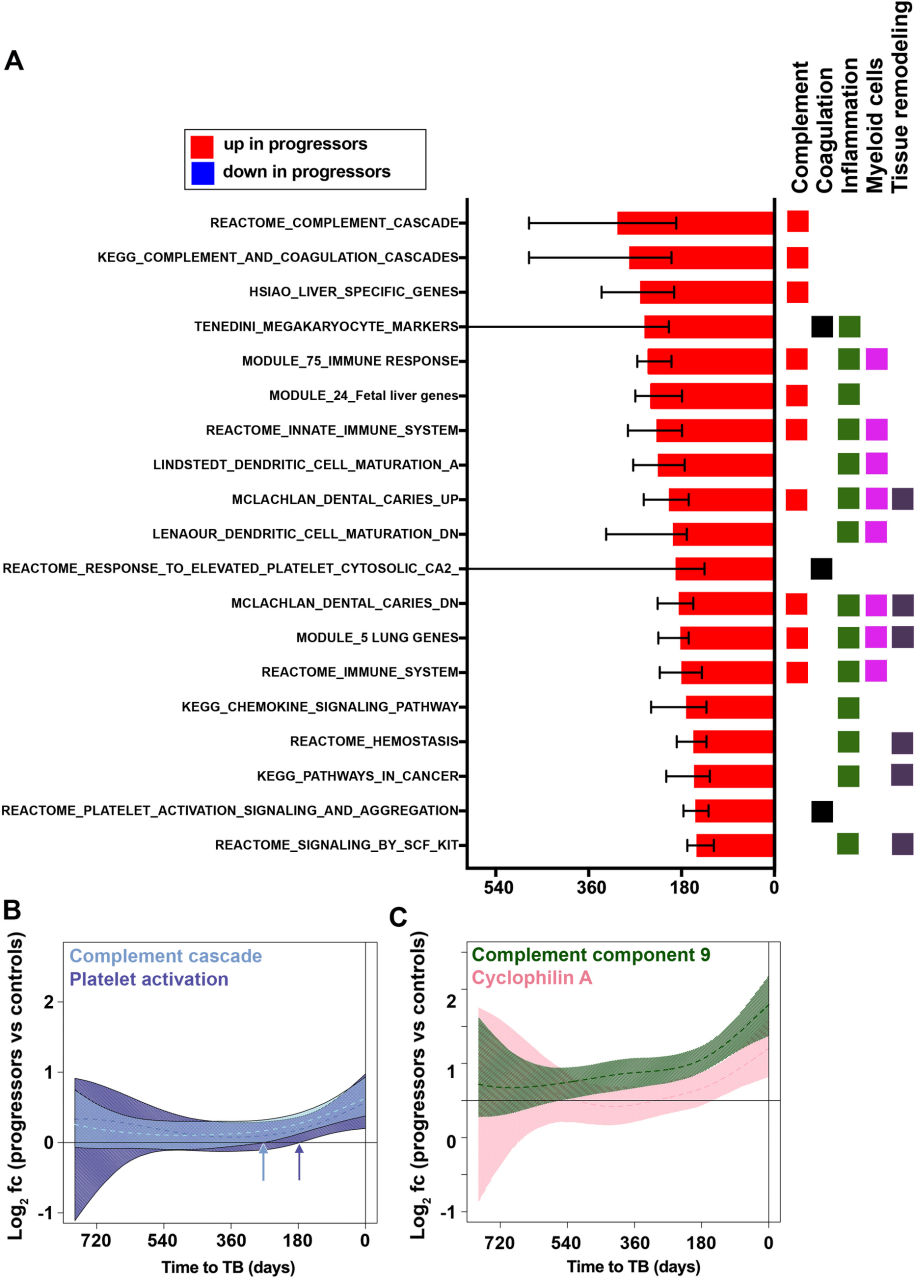
Sequential changes in plasma proteins during progression from *M*.*tb* infection to TB disease. (**A**) Gene modules, pre-defined by Reactome, KEGG and MSIGDB, and matched to the corresponding protein found to be significantly enriched in plasma from progressors, compared with controls, and ranked in descending order according to median deviation time points (indicated by bars) of proteins differentially abundant between progressors and controls. Data from 36 progressors and 104 controls were included in the analysis. Error bars denote IQR of median deviation time points of differentially abundant plasma proteins within each gene module. Assignment of each module to known immunological responses or processes or cellular subsets, according to differentially abundant proteins, is indicated by the colored squares. The full list of significantly enriched modules is in **S5 Table**. (**B**) Kinetics of complement cascade and platelet activation protein modules. Module kinetics during progression were modeled as non-linear splines (dashed lines) and 99% CI (shaded areas) were computed by performing 2000 spline fitting iterations after bootstrap resampling from the full dataset. Arrows indicate the time before TB diagnosis at which the 99% CI deviates from zero for the two modules. (**C**) Kinetics of individual proteins representing the complement cascade (complement component 9) and platelet activation (cyclophilin A) protein modules, modeled as non-linear splines and 99% CI.

Module enrichment analysis of plasma proteins revealed coordinated kinetic changes during progression, comparable to what was observed by transcriptomic analysis (**S5 Table**). Complement cascade modules (containing Complement factor I, H and B, C1s, C2, C3b, C5, C9), were up-regulated earliest during progression, compared with controls (**Fig 3A**). These changes were present at the same time as upregulation of IFN response genes shown by transcriptomic analysis. This was followed by changes in blood coagulation modules (containing coagulation factor X, fibrinogen, D-dimer, fibrinogen gamma chain, thrombospondin 1, SERPIN A1 and D1, and platelet factor 4) and by myeloid inflammation modules (containing CXCL9, CCL1, CD163, IL-6 and RANTES), which emerged after complement activation, around 200 days before TB diagnosis. Finally, several modules associated with tissue remodeling (containing MMP1, MMP9, MMP12 and tissue inhibitor of MMPs 2 and 3), hemostasis, and platelet activation emerged within 200 days before TB (**Fig 3A, 3B and 3C**). Despite the detection of many individual proteins down-regulated during TB progression (**S4 Table**) no significant enrichment for down-regulated protein modules was detected (**S5 Table**). Many proteins that significantly changed in abundance during progression could not be mapped to protein modules. Those with demonstrated importance for host defense against *M*.*tb* included granulocyte peptides neutrophil defensin 1 (HNP-1, *DEFA1*), cathelicidin (*CAMP*), beta-defensin-110 (*DEFB110*) and -131 (*DEFB113*) and neutrophil-activating peptide 2 (NAP2)–all of which were upregulated. Leukotriene A4 hydrolase (*LTA4H*), which has been implicated as an important regulator of the balance between protective and pathogenic inflammation [3,23], and NK cell products (killer cell immunoglobulin-like receptor 2DL4 (KIR2DL4) and granzyme K), were downregulated. Lastly, levels of total IgG and IgA were elevated in plasma from progressors (**S4 Table**).

To identify links between temporal changes in blood mRNA and plasma proteome data, we tested whether specific modules were over-represented within sets of proteins that exhibited progression-associated changes in abundance with kinetics that were consistent with the kinetics of key transcriptional modules. Specifically, we determined which proteins (and protein modules) showed differential abundance at deviation days that coincided with the interquartile range (IQR) of deviation days for differentially regulated genes from the ACS signature of risk of TB and the IFN response and inflammation modules (S9 **Table**). No proteins with significant enrichment (p<0.05) within defined protein modules (S5 **Table**) had deviation days that coincided with the deviation day IQR of the 16 genes within the whole blood signature of risk of TB. However, proteins that were temporally associated with the IFN response modules included CXCL10 (IP-10), STAT1 and Tryptophanyl-tRNA ligase (WARS, WRS, SYWG); and those proteins mapping to the inflammation pathway included Calgranulin C (S100A12 or EN-RAGE), alpha-1-antitrypsin (SERPINA1) and Myeloblastin or proteinase 3 (PR3), MMP9 and Ficolin-1 (FCN1) (S9 **Table**). These data provide protein-level confirmation for the finding from gene expression analyses that the IFN response precedes myeloid inflammation.

### Changes in peripheral blood cellular subsets during progression

**Fig 4:**
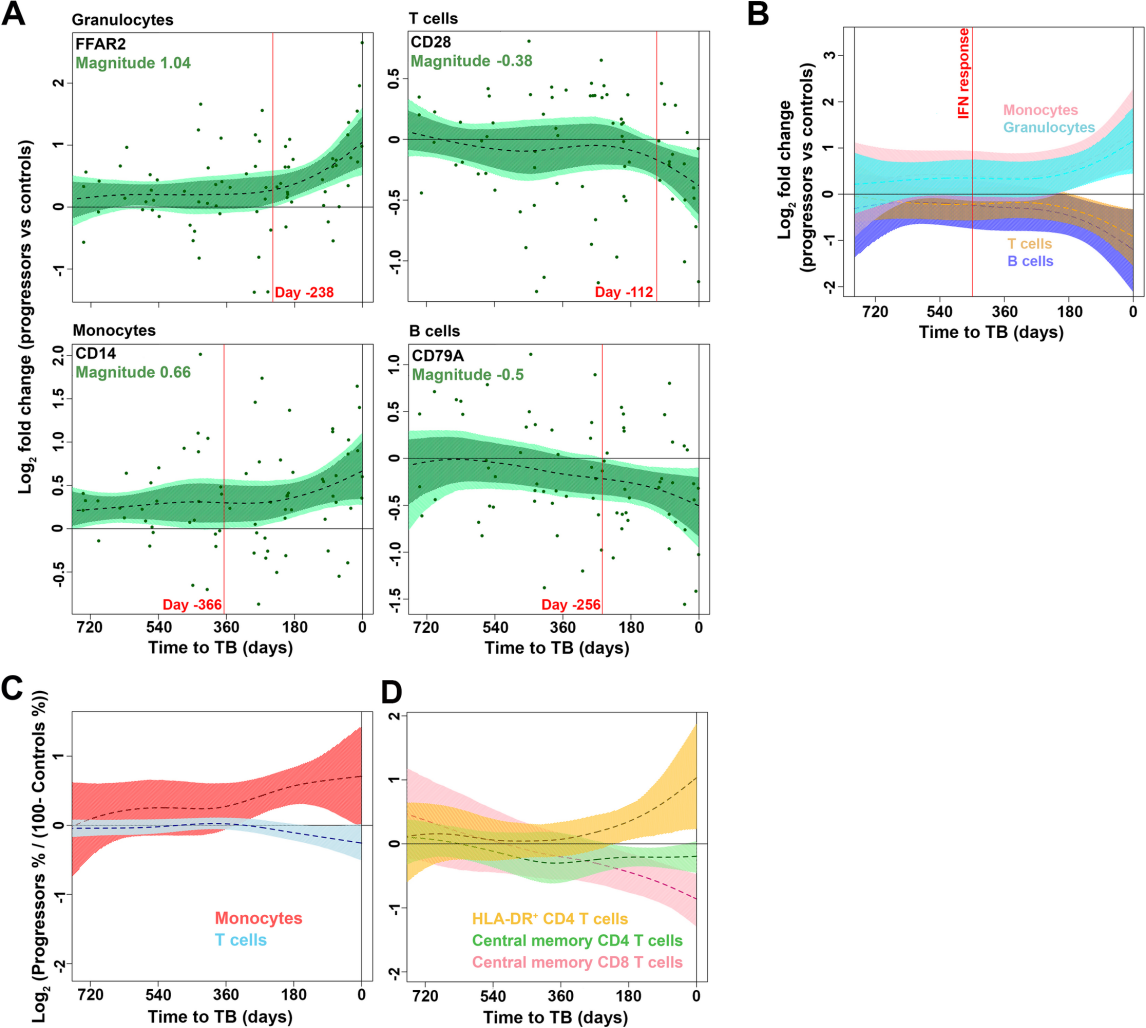
Changes in proportions of peripheral blood cell subsets during progression from infection to TB disease. (**A**) Kinetics of mRNA expression, expressed as log_2_ fold change between bin-matched progressors and controls and modeled as non-linear splines (dotted lines) for genes representing granulocytes, monocytes, T cells and B cells. Light green shading represents 99% CI and dark green shading 95% CI for the temporal trends, computed by performing 2000 spline fitting iterations after bootstrap resampling from the full dataset. The magnitude for each gene, representing the log_2_ fold change at TB diagnosis, is shown in green text. The deviation time, calculated as the time point at which the 99% CI deviates from a log_2_ fold change of 0, is indicated in red text. Data from 38 progressors and 104 controls were included in the analysis. (**B**) Temporal trends of gene modules representing granulocytes (genes with significant kinetic response from BIOCARTA_GRANULOCYTES_PATHWAY), monocytes (genes from M11.0_enriched in monocytes (II)), T cells (genes from M4.1_T-cells, M6.15_T-cells, M7.1_T cell activation (I) and M7.4_T cell activation (III)) and B cells (genes from M4.10_B-cells, M47.0_enriched in B cells (I), M47.1_enriched in B cells (II) and M69_enriched in B cells (VI)), modeled as non-linear splines during progression. The deviation time for the interferon module shown in Fig 3B is denoted by the vertical red line. (**C**) Temporal trends of relative mean (dotted lines) proportions of monocytes and T cells (**D**) or activated HLA-DR+ CD4 T cells, CCR7+CD45RA- central memory CD4 or CD8 T cells, measured by flow cytometry from cryopreserved PBMC and modeled as non-linear splines during progression. Shown are log_2_ cell proportions for progressors relative to bin-matched controls. Data from 33 progressors and 71 controls were included in the analysis. Shading denotes 99% CI computed by performing 2000 spline fitting iterations after bootstrap resampling from the full dataset.

Our transcriptomic and proteomic data highlight profound inflammatory processes during progression that are detectable more than a year before TB diagnosis. Since inflammation is known to regulate both myelopoiesis and lymphopoiesis [24], we sought to investigate changes in peripheral blood cell subsets during progression. First, we investigated kinetic changes in whole blood transcripts associated with granulocytes, monocytes, T cells and B cells. mRNA expression of FFAR2 (a representative granulocyte gene) and CD14 (a representative monocyte gene) were significantly upregulated while CD28 (a representative T cell gene) and CD79A (a representative B cell gene) were downregulated in progressors relative to controls (**Fig 4A**). Analysis of gene modules representing these four blood cell subsets supported these changes (**Fig 4B**) and suggested that modulation of myeloid and lymphoid compartments during TB progression was secondary to the up-regulation of the genes in the whole blood signature of risk for TB and the induction of IFN response genes in general, which markedly preceded changes in peripheral blood cellularity. To confirm these data, we enumerated proportions of major blood cell subsets by flow cytometry (**S3A Fig**). Within 200 days of TB diagnosis relative proportions of CD14+ monocytes were significantly increased while CD3+ T cells were depleted in progressors, relative to controls (**Fig 4C**). These changes were accompanied by T cell activation in progressors, indicated by elevated expression of HLA-DR on CD4 T cells, with concomitant decreases in relative proportions of CD45RA-CCR7+ central memory CD4 and CD8 T cells (**Fig 4C**).

### T cell functional capacity is modulated during progression to TB

**Fig 5:**
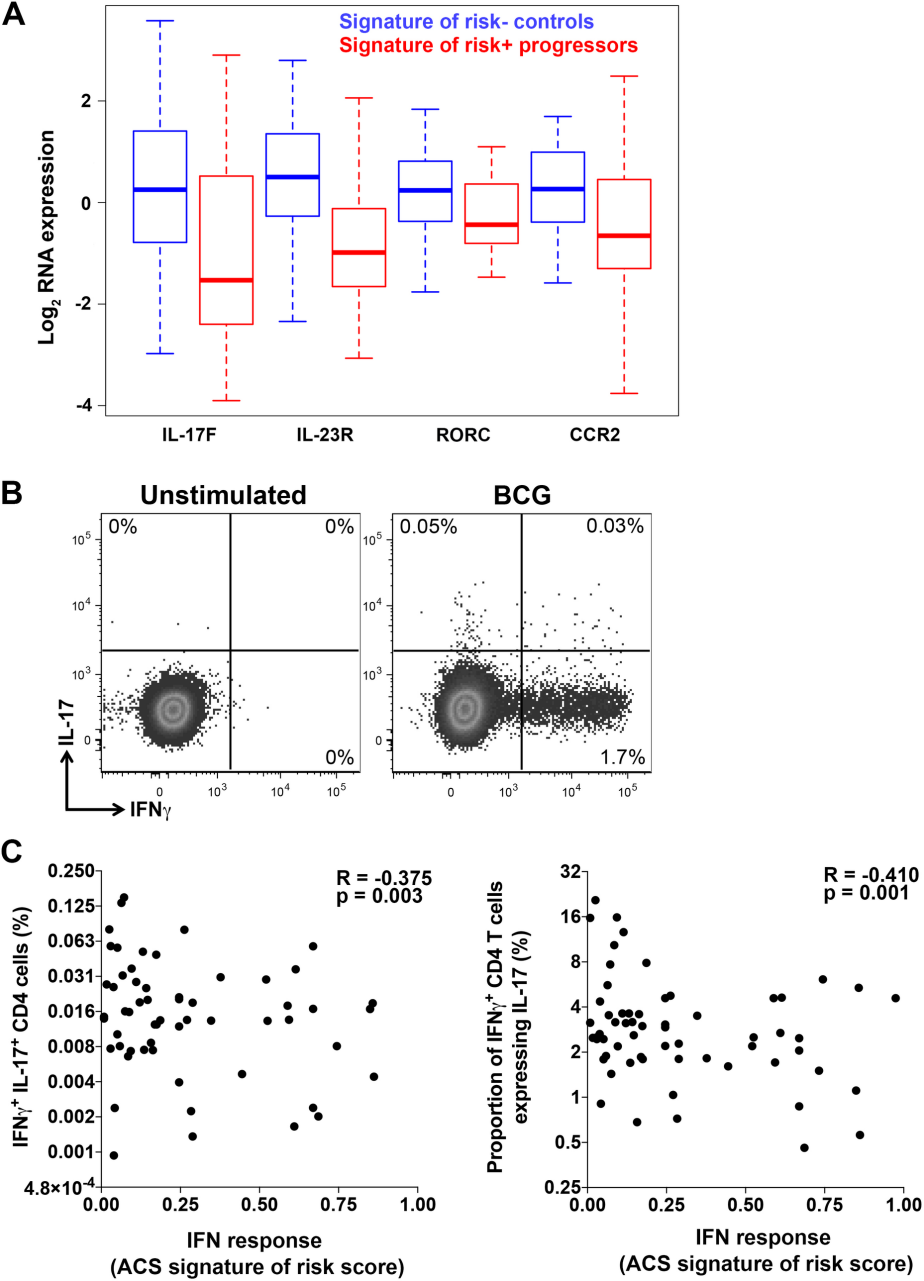
Changes in T cell function associated with whole blood IFN responses in progressors. (**A**) Differentially expressed Th17-associated mRNA transcripts in sorted T cells from progressors with positive ACS signature of risk of TB compared with controls with negative signature of risk of TB. T cells were sorted after stimulation of PBMC with medium alone or peptide pools and data from these stimulation conditions were combined for analysis (see methods). Data from 31 progressors (138 progressor samples were signature-positive, 67 were negative) and 90 controls (299 control samples were signature-negative, 40 were positive) were included in the analysis and time to TB was not considered. Representative genes significantly enriched in the Th17 module by modular analysis, at a p-value < 0.05 and an FDR <0.2, are shown. The full set of differentially expressed T cell genes is in **S6 Table** and gene modules enriched in genes differentially expressed between progressors with positive ACS signature of risk of TB and controls with negative ACS signature of risk of TB are listed in **S7 Table**. (**B**) Flow cytometry plots depicting CD4 T cells co-expressing IFNγ and IL-17 after stimulation of whole blood with BCG or medium (unstimulated) from an adult in the BCG revaccination study. Shown is a representative sample taken 3 weeks after BCG-revaccination. (**C**) Associations between cytokine expressing CD4 T cells after stimulation of whole blood with BCG or medium (unstimulated) and the ACS signature of risk of TB (COR score), in adults from the BCG revaccination study. Type I/II IFN response was measured by the ACS signature of risk of TB. Shown are frequencies of BCG-specific CD4 T cells co-expressing IFNγ and IL-17 and relative proportions of BCG-specific IFNγ^+^ CD4 T cells co-expressing IL-17. Spearman R and p-values are shown in each plot.

T cells and specifically antigen-specific IFNγ-expressing CD4 T cells are necessary for successful control of *M*.*tb* infection [25,26]. *In vitro* studies have shown that type I IFNs can inhibit the macrophage antimycobacterial response mediated by IFNγ [27]. To determine if expression of the whole blood signature of risk for TB and IFN response gene module is associated with concomitant functional changes to T cells, we performed RNA-Seq transcriptome profiling of T cells sorted from adolescent progressor and control PBMCs. Comparing transcriptomes of T cells obtained from TB progressors that expressed the whole blood signature of risk to those from controls that did not express the whole blood signature of risk revealed 277 genes that were significantly differentially expressed between the populations (**S6 Table**). Modular analysis showed that genes associated with hypoxia response and cell cycle were prominent amongst genes that were expressed at lower and higher levels, respectively, in progressor T cells (**S2B and S2C Fig** and **S7 Table**). Another striking result was that the Th17-associated genes IL-17F, IL-23R, RORC and CCR2 were expressed at lower levels in T cells from progressors expressing the whole blood signature of risk for TB (**Fig 5A**). This suggests that induction of Th17 responses may be inhibited in progressors with high ISG expression in the blood—a result that may have implications for vaccination in persons exposed to *M*.*tb*.

### Impaired Th17 function after BCG revaccination in individuals with high ISG expression

To test the hypothesis that expression of the ISGs comprising the signature of risk for TB in whole blood is associated with a concomitant suppression of Th17 function, we analyzed T cell responses to Bacille Calmette-Guerin BCG revaccination in an independent cohort of South African adults with latent *M*.*tb* infection [17]. This cohort exhibited a broad range of scores for the whole blood signature of risk for TB, spanning low to high IFN response magnitudes before BCG administration. Three weeks after BCG administration BCG-specific CD4 T cells expressing Th1 cytokines and IL-17 were enumerated by flow cytometry (**S3B Fig and Fig 5B**). Frequencies of BCG-specific CD4 T cells that co-expressed IFNγ and IL-17 as well as relative proportions of BCG-specific IFNγ^+^ CD4 T cells that co-expressed IL-17 were inversely correlated with whole blood expression of the signature of risk for TB (**Fig 5C**). By contrast, frequencies of BCG-specific CD4 T cells expressing any cytokine, co-expressing IFNγ and TNF, or relative proportions of BCG-specific IFNγ^+^ CD4 T cells that co-expressed TNF, were not associated with the whole blood expression of the signature of risk for TB, and neither were frequencies of total IL-17^+^ CD4 T cells (**S4 Fig**). These data suggest that underlying systemic inflammatory perturbations that are associated with risk of TB progression, as indicated by the signature of TB risk score, may interfere with induction or maintenance of antigen-specific Th17 cells after vaccination.

## Discussion

We report orchestrated, sequential changes in blood mRNA, soluble protein and cellular responses during the transition from asymptomatic *M*.*tb* infection to active pulmonary TB disease in a prospective, longitudinal cohort of adolescents. Particularly striking was that these changes exhibited a spectrum of kinetics, with a minority of responses exhibiting detectable differences 1–2 years before diagnosis, and the largest suite of differences between progressors and controls being observed most proximal to TB disease. These data suggest that TB progression is a slow but steady transition from an immunologically quiescent state, via nondiscrete progressive stages of inflammatory perturbation to the highly inflammatory, clinical manifestations (fever, cough, hemoptysis and weight loss) of microbiologically confirmed, active TB disease. Our results suggest that an intermediate *M*.*tb* infection state that appears consistent with incipient or subclinical TB in individuals with no other signs of TB disease, can be revealed with blood biomarkers, such as the whole blood signature of risk for TB progression [15], and specific elevation of IFN response gene modules and activation of the complement cascade. Our recent report of infants with QFT conversion values which exceeded > 4IU/mL of IFN-γ and who were at exceptionally high risk of TB disease within 6 months of QFT conversion [28], support this finding. This is also consistent with the description of subclinical TB disease recently reported in a proportion of asymptomatic, antiretroviral therapy naïve, HIV-infected individuals with latent *M*.*tb* infection, who presented with pulmonary abnormalities on combined positron emission and computed tomography (PET-CT) [29]. Of note, four of the ten individuals with evidence of subclinical disease developed symptomatic active TB within 6 months, suggesting that such individuals are progressing towards clinical disease.

Our study lends a timeline to the different immunological stages of TB progression. The first of these, detected up to 18 months before TB diagnosis, included elevated expression of the signature of risk genes themselves and expression of IFN responses genes and complement activation more broadly. Although we did not detect elevated levels of soluble IFNα, β or IFNγ proteins themselves in plasma from progressors, as was also observed in a proteomic study of TB disease [22], it is well-established that expression of type I IFNs by *M*.*tb*-infected macrophages can be activated by bacterial DNA via STING signaling following binding to cGAS [30–32]. Further, *M*.*tb*-induced mitochondrial stress and abundance of mitochondrial DNA in the cytosol of infected macrophages were also recently shown to drive IFNβ expression [33]. These papers suggest that the IFN response signature may be directly activated by *M*.*tb* bacilli. However, our data do not reveal whether the IFN response cascade is induced by type I or II IFNs. Other well-known inducers of type I IFNs, such as viral infections, may also underlie the IFN response and even contribute to a higher risk of progression to active TB [25,34]. For example, influenza infection has been shown to reduce host resistance to *M*.*tb* in mice [35].

Plasma protein levels of the established interferon (type I and/or type II)-induced proteins CXCL10, STAT1 and Tryptophanyl-tRNA ligase were up-regulated with kinetics that were consistent with the up-regulation kinetics for the transcriptomic IFN response. Further, increases in protein levels of the inflammatory proteins Calgranulin C, alpha-1-antitrypsin, Myeloblastin, MMP9 and Ficolin-1 were temporally associated with upregulation of the inflammation transcriptomic module. Calgranulin C, and the serine proteases alpha-1-antitrypsin and Myeloblastin are involved in the neutrophil response and have been previously implicated in mycobacteria-induced inflammation [36]. Interestingly, the *M*.*tb* secreted proteins Ag85 and ESAT-6 have been shown to induce expression of Ficolin-1 [37] and MMP-9 [38], respectively. Ficolin-1 is a pattern recognition molecule that can activate complement via the lectin pathway [39] and MMP-9 plays an important role in macrophage recruitment and granuloma establishment after *M*.*tb* infection [38]. These data further support the interpretation that the inflammatory responses observed during progression may be directly triggered by increased bacterial replication.

We also observed a strong complement activation signal during the very early stages of TB progression, which coincided with elevation of the IFN response. In light of the well-described roles of complement components, such as C1, C3 and C4, in *M*.*tb* recognition and phagocytosis [40,41], these data are also consistent with host innate sensing of *M*.*tb* in progressors, either via increased pathogen load or greater access to the bacterium. We were not able to establish from our data whether complement was activated through a particular pathway. Mycobacteria are able to activate complement through the antibody-dependent and antibody-independent classical pathways, alternative pathway activation and the lectin pathway [39,42]. We observed upregulation of components consistent with activation of the classical pathway via C1q, while levels of total IgG and IgA were also upregulated in progressors. Differential abundance of mannose-binding lectin (MBL) and ficolin-1 proteins in plasma also implicate activation of the lectin pathway. Finally, while a number of proteins involved in activation of the alternative pathway were elevated in progressors, these can also indicate complement activation by any pathway [39]. Complement may also be activated by pro-inflammatory stimuli and, in turn, components of the complement cascade are known regulators of inflammation [39]. Our data thus highlight that the complex interplay between *M*.*tb*, inflammation, antibody responses and complement activation needs greater exploration.

Secondary to these IFN responses and complement activation during progression, increased myeloid cell inflammation, platelet activation and blood coagulation, with concurrent enrichment of peripheral blood monocytes and other myeloid cells were observed 12 to 6 months before disease diagnosis. Finally, within the most proximal 6 months before TB disease, changes in lymphocytes, including suppressed T and B cells and enrichment in neutrophils, were detected. The latter coincided with activation of tissue remodeling pathways that included elevated expression of several MMPs. High MMP concentrations correlate with lung immunopathology in TB disease, demonstrating the role of MMPs as effectors of matrix destruction in TB [43,44]. We speculate that our data suggest that breakdown of the extracellular lung matrix may occur months before clinical TB manifestation. If so, intervention during early stages of progression may allow prevention of pulmonary caseation, necrosis and cavitation, which are associated with poor treatment outcome [45].

Our findings are consistent with those of a recent study of blood transcriptional signatures in the cynomolgus macaque model of TB [46]. This study revealed elevated IFN responses, myeloid inflammation, complement activation and coagulation/platelet and myeloid lineage pathways, and decreased T cell, B cell and cytotoxicity pathways, in *M*.*tb*-infected macaques prior to clinical manifestation and divergence into active and latent TB. Further, macaques that ultimately developed active TB had elevated expression of IFN response signatures and lower expression of lymphoid cell gene modules by 30 days post-*M*.*tb* infection, compared with animals that maintained latent infection [46].

Our findings also complement those of numerous investigators who described transcriptomic signatures of active TB disease, characterized by highly elevated expression of ISGs and upregulated myeloid inflammation, neutrophil and FC receptor/complement pathways [11–14]. Such inflammatory signatures were previously also reported in a small proportion of apparently healthy individuals and led to the recognition that asymptomatic infection with *M*.*tb*, traditionally referred to as latent TB, exists as a spectrum that ranges from quiescence to subclinical TB disease [16]. Our results support this interpretation and add a timeline to the transition through apparent stages within the spectrum. It should be noted that transition from quiescent infection, through incipient and subclinical TB to active pulmonary disease in different individuals was highly heterogeneous. A limitation of our study is that the time of exposure and/or *M*.*tb* infection in most progressors and controls was unknown, precluding interpretation of the events that precede establishment of *M*.*tb* infection.

Finally, our T cell transcriptomic results demonstrate that progression was associated with modulation of the functional states of T cells, particularly suppressed expression of genes associated with the Th17 compartment [47,48] in progressors that expressed the whole blood signature of risk for TB. Systemic expression of IFN response genes that comprise the signature of risk occurred concomitantly with Th17 inhibition. A negative correlation between type I IFN responses and Th17 responses has been reported in other systems [49–51]. This link between high expression of ISG as measured by the signature of risk of TB, and an alteration in T cell functional capacity was confirmed by analysis of an independent cohort. In South African adults who expressed the signature for risk of TB in whole blood, BCG revaccination induced significantly lower frequencies of IFNγ^+^IL-17^+^ and decreased proportions of IL-17-expressing IFNγ^+^ CD4 T cells. A major implication of this result is that immune responsiveness to vaccination may be modulated by the inflammatory milieu associated with progression to active TB, and even other immune modulations that result in systemic persistent expression of IFN response genes, such as viral infections. Further research is required to dissect the mechanistic link between inflammatory and cellular events that may underlie this observation, and to understand the true implications of this finding.

Our study shows that sequential inflammatory dynamics precede TB disease manifestation characterized by specific alterations in blood transcriptomic, proteomic and cellular signatures. The detectable immunological and tissue remodeling perturbations observed in progressors suggest that new vaccination and drug treatment strategies and/or host-directed therapies may be required to control *M*.*tb* in persons with subclinical TB disease, while identifying potential targets (and potential targets to avoid) for successful interventional approaches to prevent progression to active TB. Careful investigation of this phenomenon is warranted.

## Materials and methods

### The adolescent cohort study

We analyzed samples from *M*.*tb*-infected participants of the South African Adolescent Cohort Study (ACS), previously evaluated to identify and validate the signature for risk of TB [15].

Briefly, 6,363 healthy adolescents, aged 12–18 years, were enrolled between July 2005 and April 2007 and follow-up was completed by February 2009. Approximately half of the adolescents were evaluated at enrollment and every 6 months during 2 years follow-up; the other half was evaluated at baseline and at 2 years. At enrollment and at each visit, clinical data were collected, 2.5mL blood was collected directly into PAXgene blood RNA tubes (PreAnalytiX) and blood was collected in Cell Preparation Tubes (BD Biosciences) and peripheral blood mononuclear cells and plasma were isolated using density gradient centrifugation.

Only adolescents with *M*.*tb* infection at enrollment, or those who developed active TB disease more than 6 months after *M*.*tb* infection was first detected were included in our analyses, diagnosed by a positive QFT (Qiagen; >0.35 IU/mL) and/or a positive TST (0.1mL dose of Purified Protein Derivative RT-23, 2-TU, Staten Serum Institute; >10mm). According to South African policy, QFT and/or TST positive adolescents were not given therapy to prevent tuberculosis disease. Progressors were adolescents who developed active TB disease during follow-up, defined as intrathoracic disease, with either two sputum smears positive for acid-fast bacilli or one positive sputum culture confirmed as *M*.*tb* complex (mycobacterial growth indicator tube, BD BioSciences). For each progressor, two matched controls who remained healthy during follow-up were selected and matched by age at enrolment, gender, ethnicity, school of attendance, and presence or absence of prior episodes of tuberculosis disease (**Table 1 and Fig 1**). Participants were excluded if they developed tuberculosis disease within 6 months of enrollment or QFT and/or TST conversion, to exclude early asymptomatic disease that could have been present at the time of evaluation, or if they were HIV infected. Participants with diagnosed or suspected tuberculosis disease were referred to a study-independent public health physician for treatment according to national tuberculosis control programs of South Africa.

### Adult trial of BCG revaccination

Effects of IFN responses on T cell responses after BCG revaccination were assessed in *M*.*tb*-infected adults who participated in a previous trial of BCG revaccination [17,52]. Briefly, we recruited healthy 18 to 40 year old South African adults, who were strongly TST positive (≥ 15mm induration when tested with PPD RT-23); HIV-seronegative; received BCG at birth and had a visible BCG scar. In this phase I trial, participants, recruited from the population of Worcester in the Western Cape, South Africa were randomized in parallel into two groups in a 1:1 ratio as previously described [17,52]. Participants in the first group were observed for 7 months, then vaccinated with BCG, and subsequently treated with isoniazid (INH) 6 months later (Observation-BCG-INH). Participants in the second group received a course of 6 months of INH within a maximum period of 7 months, followed by BCG vaccination (INH-BCG-Observation). Danish strain 1331 BCG Vaccine SSI (Statens Serum Institut, Copenhagen, Denmark), the BCG vaccine used in the South African national immunization program and one of the most widely administered BCG vaccines worldwide, was administered intradermally at an adult dose of 2 to 8 x 10^5^ CFUs. INH (Westward Pharmaceutical Corporation, Eatontown, NJ, USA) was administered daily at 5mg/kg rounded up to the nearest 100mg (maximum dose 300 mg/day), and INH adherence was monitored by pill counts at clinic visits and random urine INH metabolite testing (18). All participants provided written, informed consent. Whole blood was collected in PAXGene tubes and in Sodium-Heparin tubes from participants and processed within 45 minutes of phlebotomy, as previously described (19), at enrollment, 1 month after isozianid preventive therapy initiation, at BCG vaccination, at 3 and 5 weeks, and 1 year post-vaccination. RNA was isolated from PAXGene tubes as described above. Heparinized blood was stimulated and processed for measurement of T cell responses by whole blood intracellular cytokine staining (WB-ICS) assay, as previously described [17]. The signature of risk of TB was measured in samples collected before BCG re-vaccination and the functionality of the T cell response to BCG revaccination was measured 3 weeks after vaccination.

### Ethics statement

ACS study protocol, including sample collection, utilization and analyses, were approved by the Human Research Ethics Committee of the Faculty of Health Sciences, University of Cape Town. Written informed consent was obtained from parents or legal guardians, and written informed assent from each adolescent.

The BCG revaccination trial protocol, including sample collection, utilization and analyses, were approved by the Medicines Control Council (MCC) of South Africa, Human Research Ethics Committee (HREC) of the University of Cape Town and the University Hospitals Case Medical Center institutional review board. The trial was registered on ClinicalTrials.gov (NCT01119521). Written informed consent was obtained from all participants.

### RNA sequencing (RNA-Seq) and analyses

Generation of the whole blood RNA-Seq data was previously described [15]. RNA was extracted from PAXgene tubes, globin transcripts were depleted and (GlobinClear, Life Technologies) cDNA libraries were prepared using Illumina mRNA-Seq Sample Prep Kit. RNA-Seq was performed by Expression Analysis Inc., at 30 million 50bp paired-end reads, on Illumina HiSeq-2000 sequencers.

For monocytes and T cells, RNA was extracted from cells left unstimulated, stimulated with *M*.*tb* antigens (ESAT-6/CFP-10 or Ag85A/B; T cells), or infected with *M*.*tb* (monocytes and T cells). RNA-Seq was performed by Expression Analysis Inc. as described [15] or (Unstimulated and *M*.*tb* antigen-stimulated T cells) Beijing Genomics Institute (Shenzen, China) after performing amplification (Clontech SMARTer Universal Low Input RNA Kit). RNA-Seq alignment, QC, and gene-level summarization for whole blood, monocytes, and T cells were also performed as described [15].

Whole blood, monocyte and CD4 T cell RNA-Seq data was aligned to the hg19 human genome using gsnap [53] as in the original study [15]. Normalized gene-level expression estimates were derived from mapped read pairs following the procedure implemented previously [54]. Briefly, mapped read pairs were assigned to genes by collapsing all transcripts into a single gene model and counting the number of reads that fully overlap the resulting exons using htseq (v. 0.6.0) [55], with strict intersection and including strand information. Gene models for protein-coding genes were downloaded from Ensembl (GRCh37.74). Reads that mapped to multiple locations were only counted once and those mapping to ambiguous regions were excluded. Log_2_-transformed values of counts normalized by adjusted library counts were computed using the cpm function of the edgeR package [56]. For monocyte and CD4 T cell transcriptomic analyses, both RNA-Seq and qRT-PCR measurements of the ACS signature of risk of TB score (**S1 Table**) were used to classify samples as positive (> 0.6 in both RNA-Seq and qRT-PCR) or negative (< 0.4 in both RNA-Seq and qRT-PCR), to ensure robust classification.

### Plasma proteomic analysis

Cryopreserved plasma samples collected from BD Vacutainer Cell Preparation Tubes with Sodium Heparin (BD Biosciences) were analysed by using SOMAscan Version 3+ 3000plex assay, a multiplexed modified DNA aptamer array that quantifies 3000 proteins at 3 different plasma dilutions, as reported previously [21,22]. Data from all samples were log_2_ transformed, normalized and calibrated using standard hybridization and calibration procedures.

### Temporal analysis of transcriptional and proteomic data

Prospective RNA-Seq data of progressors were realigned to the time point at which active tuberculosis was diagnosed (TimeToDiagnosis), as described in [15], thereby synchronizing the cohort with respect to outcome. Differences in gene-level mRNA expression or protein concentrations between each progressor sample and the average of demographically matched control samples were computed using the published ACS metadata (**S1 Table** [15]). TimeToDiagnosis values were assigned to each sample according to the original definitions. The log_2_ fold change values between progressor and control biomarkers were modeled as a nonlinear function of TimeToDiagnosis for the entire population using the smooth.spline function in R with three degrees of freedom. Ninety-nine percent confidence intervals for the temporal trends were computed by performing 2000 iterations of spline fitting after bootstrap resampling from the full dataset.

### Transcriptomic analyses of sorted T cells and monocytes

Cyropreserved PBMC from progressors and controls were thawed and used for *M*.*tb* antigen stimulation experiments to sort monocytes and T cells for transcriptomic analyses. Specifically, CD14+ monocytes were sorted by positive selection using Miltenyi CD14 microbeads on an AutoMACS Pro to a purity of >90% (verified by flow cytometry). Two x 10^5^ sorted monocytes were subsequently stimulated with 2x10^6^ CFU/ml live H37Rv *M*.*tb* in 0.5mL final volume, or left unstimulated for 6 h at 37°C.

Similarly, for analyses of T cells, thawed PBMC were rested at 37°C for 4–6 hours, and 1x10^6^ live PBMC were stimulated with 1x10^6^ CFU/ml live H37Rv in 0.5 mL final volume, at 37°C for 12 hr, or with pools of 15mer peptides overlapping by 10 amino acids (1μg/ml/peptide), of ESAT-6 and CFP-10, or Ag85A and Ag85B. Stimulation in media with 0.27% DMSO served as the negative control. Anti-CD28 and anti-CD49d co-stimulatory antibodies (1μg/ml, BD Biosciences) were added to the peptide pool stimulated and negative control conditions. After stimulation at 37°C for 12 hours, T cells were purified from PBMC by negative selection using Miltenyi Pan-T cell isolation kit on an AutoMACS Pro (for peptide and negative controls), or manually using MACS columns under BSL-3 conditions for *M*.*tb*-stimulated samples to a purity of >99% (verified by flow cytometry). Purified T cells were lysed in RNeasy RLT buffer (QIAgen) while purified monocytes and *M*.*tb*-stimulated T cells were lysed in PrimeStore MTM buffer (Longhorn Vaccines and Diagnostics). RNA was extracted from sorted cell subsets using RNeasy Plus Micro kit (QIAgen) and subjected to RNA sequencing as described above.

### Module enrichment analysis

To test for coordinated changes in functionally-associated genes and proteins enrichment analysis was performed using predefined module definitions [18,19,57]. Module enrichments were performed by treating the timepoint of deviation between progressors and controls, defined as the day before TB diagnosis on which the 99% CI deviated from a log_2_ fold change of 0, for each gene/protein as a predictor. Only modules with enrichment (adjusted p-value < 0.05) and more than 9 genes or proteins with kinetic response were considered to have a significant kinetic response during progression.

### Flow cytometry

For the progressor and controls analyses, PBMC were stained anti-CCR7-PE (150503) at 37°C for 20 min and thereafter at 4°C for 30 mins with a cocktail of the following antibodies: CD45-FITC (2D1), CD11c-PerCP-Cy5.5 (Bu15), CD19-PE-Cy7 (SJ25C1), CD45RA-APC (550855), HLA-DR-AF700 (L243, BioLegend), CD14-Qdot585/605 (TuK4), CD3-V450 (UCHT1), CD8-Qdor655 (3B5, Invitrogen), CD4-V500 (RPA-T4) and the live/dead dye, 7AAD (ViaProbe). All antibodies and dyes were from BD Biosciences, unless otherwise indicated. Cells were acquired on a BD LSRFortessa flow cytometer and analysed using FlowJo (v9.2). Dead cells and doublets were excluded before cell subset proportions were computed. The gating strategy on a representative donor sample is shown in **S1A Fig.**

For the BCG revaccination analyses, cryopreserved, fixed whole blood samples were thawed and stained at 4°C in BD perm/wash buffer (BD) with CD3-ECD (UCHT1, Beckman-Coulter), CD4-Qdot605 (S3.5, Invitrogen), CD8-APC-H7 (SK1), TCR-γδ-BV421 (B1), CD56-BV711 (HCD56, Biolegend), IFNγ-Alexa700 (B27), TNFα-PECy7 (MAb11, eBioscience), IL-2-APC (MQ1-17H2), IL-17-Alexa488 (N49-653), IL-22-PE (IC7821P, R&D Systems). All antibodies and dyes were from BD Biosciences, unless otherwise indicated. Cells were acquired on a BD LSRFortessa flow cytometer and analysed using FlowJo (v9.2). The gating strategy on a representative donor sample is shown in **S1B Fig.**

### Accession numbers

Datasets are available in the online appendix, or for the RNA-Seq data, as follows:

Whole blood transcriptomes: GSE79362

T cell and monocyte transcriptomes: GSE103147

## Supporting information

S1 Fig Selected genes and proteins found to be significantly abundant between progressors and controls. (A) Kinetics of mRNA expression over time, expressed as log_2_ fold change between bin-matched progressors and controls and modeled as non-linear splines (dotted lines) for two representative interferon response genes and two representative inflammation genes. Light green shading represents 99% CI and dark green shading 95% CI for the temporal trends, computed by performing 2000 spline fitting iterations after bootstrap resampling from the full dataset. The relative difference in magnitude for each gene, representing the log_2_ fold change at TB diagnosis, is shown in green text. The deviation time, calculated as the time point at which the 99% CI deviates from a log_2_ fold change of 0, is indicated in red text. (B) Kinetics of plasma protein abundance over time, expressed as log_2_ fold change between bin-matched progressors and controls and modeled as non-linear splines (dotted lines) for proteins representative of the major response pathways during progression. Light purple shading represents 99% CI and dark purple shading 95% CI for the temporal trends, computed by performing 2000 spline fitting iterations after bootstrap resampling from the full dataset. The relative difference in magnitude for each protein, representing the log_2_ fold change at TB diagnosis, is shown in purple text. The deviation time, calculated as the time point at which the 99% CI deviates from a log_2_ fold change of 0, is indicated in red text.
(TIF)

S2 Fig Transcriptomic signatures in monocytes and T cells associated with whole blood IFN responses. (**A**) Levels of selected mRNA transcripts of genes found to be differentially expressed in sorted monocytes from progressors with positive ACS signature of risk of TB, indicating an IFN response, and controls with negative ACS signature of risk of TB. Data from 31 progressors (122 progressor samples were signature-positive, 44 were negative) and 90 controls (236 control samples were signature-negative, 28 were positive) were included in the analysis and time to TB was not considered. A total of 89 genes were differentially expressed; the full set is in **S8 Table**. The gene modules enriched in genes differentially expressed between progressors with positive ACS signature of risk of TB and controls with negative ACS signature of risk of TB are listed in **S8 Table**. (**B, C**) Differentially expressed mRNA transcripts in sorted T cells from progressors with positive ACS signature of risk of TB, indicating IFN responses, and controls with negative ACS signature of risk of TB. Data from 31 progressors (138 progressor samples were signature-positive, 67 were negative) and 90 controls (299 control samples were signature-negative, 40 were positive) were included in the analysis and time to TB was not considered. Representative genes significantly enriched in the hypoxia (**B**) and cell cycle (**C**) modules by modular analysis, at a p-value < 0.01 and an FDR <0.3, are shown. The full set of differentially expressed T cell genes is in **S6 Table** and gene modules enriched in genes differentially expressed between progressors with positive ACS signature of risk of TB and controls with negative ACS signature of risk of TB are listed in **S7 Table**.
(TIF)

S3 Fig Flow cytometry gating strategies. (A) Gating strategy used to measure proportions of myeloid and lymphoid cell populations in PBMC from adolescent progressors and controls. The figure shows a representative sample from an adolescent. The numbering above each plot indicates the order of gating. (B) Gating strategy used to measure frequencies of BCG-specific CD4 T cells expressing cytokines by intracellular cytokine staining assays from cryopreserved, stimulated whole blood. The figure shows a representative sample collected 3 weeks after BCG revaccination from a single donor. The sequence for gating is indicated by the numbering above the plots. Numbers within each plot indicates the proportion of cells falling into the relevant gates.
(TIF)

S4 Fig Associations between cytokine expressing CD4 T cells after stimulation of whole blood with BCG or medium (unstimulated) and the ACS signature of risk of TB (COR score), in 61 adults from the BCG revaccination study. Type I/II IFN response was measured by the ACS signature of risk of TB. Shown are frequencies of total cytokine^+^ BCG-specific CD4 T cells, cells co-expressing IFNγ and TNF, relative proportions of BCG-specific IFNγ^+^ CD4 T cells co-expressing TNF and frequencies of total IL-17^+^ CD4 T cells. Spearman R and p-values are shown in each plot.
(TIF)

S1 Table Metadata for the adolescent cohort study (ACS) progressors and controls, listed by sampling time point. (XLSX)

S2 Table List of whole blood genes with significant splines. (XLSX)

S3 Table List of gene modules enriched in genes with significant splines. 3 modules sets, Chaussabel, BTM and MSIGDB modules, were considered in this analysis.
(XLSX)

S4 Table List of proteins with significant splines. (XLSX)

S5 Table List of gene modules enriched in proteins (matched to their genes) with significant splines. 3 modules sets, Chaussabel, BTM and MSIGDB modules, were considered in this analysis.
(XLSX)

S6 Table Genes regulated in ACS signature of risk positive progressors vs ACS signature of risk negative controls in sorted T cells. T cells that were unstimulated or stimulated with Ag85A/B and ESAT-6/CFP-10 peptide pools were pooled in this analysis.
(XLSX)

S7 Table List of gene modules enriched in genes differentially expressed by sorted T cells between ACS signature of risk positive progressors vs ACS signature of risk negative controls. (XLSX)

S8 Table Genes regulated in ACS signature of risk positive progressors vs ACS signature of risk negative controls in unstimulated and Mtb (H37Rv) stimulated monocytes. (XLSX)

S9 Table Proteins and enriched protein modules with differential abundances and deviation days that fall within the deviation day IQRs of genes within the ACS signature of risk of TB, Interferon and Inflammation modules. (XLSX)

S1 Methods Supplementary methods. (DOCX)
