# Supplementary figures and images for: Sequential inflammatory processes define human progression from M. tuberculosis infection to tuberculosis disease
Source: PLoS Pathog. 2017 Nov 16;13(11):e1006687. doi: 10.1371/journal.ppat.1006687 (PMC5689825; doi:10.1371/journal.ppat.1006687)

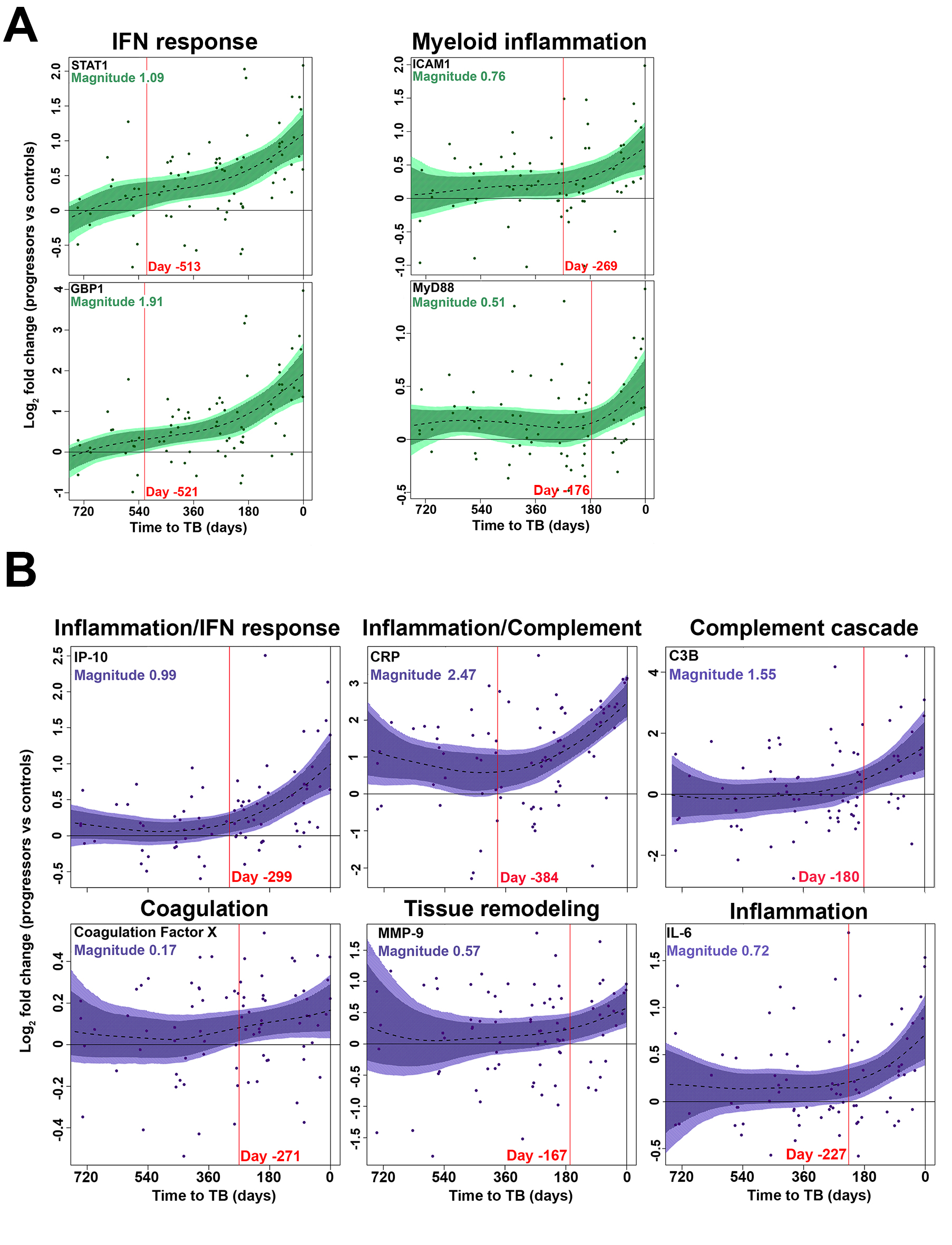

Supplement: S1 Fig — (A) Kinetics of mRNA expression over time, expressed as log2 fold change between bin-matched progressors and controls and modeled as non-linear splines (dotted lines) for two representative interferon response genes and two representative inflammation genes. Light green shading represents 99% CI and dark green shading 95% CI for the temporal trends, computed by performing 2000 spline fitting iterations after bootstrap resampling from the full dataset. The relative difference in magnitude for each gene, representing the log2 fold change at TB diagnosis, is shown in green text. The deviation time, calculated as the time point at which the 99% CI deviates from a log2 fold change of 0, is indicated in red text. (B) Kinetics of plasma protein abundance over time, expressed as log2 fold change between bin-matched progressors and controls and modeled as non-linear splines (dotted lines) for proteins representative of the major response pathways during progression. Light purple shading represents 99% CI and dark purple shading 95% CI for the temporal trends, computed by performing 2000 spline fitting iterations after bootstrap resampling from the full dataset. The relative difference in magnitude for each protein, representing the log2 fold change at TB diagnosis, is shown in purple text. The deviation time, calculated as the time point at which the 99% CI deviates from a log2 fold change of 0, is indicated in red text. (TIF) [file ppat.1006687.s001.tif]

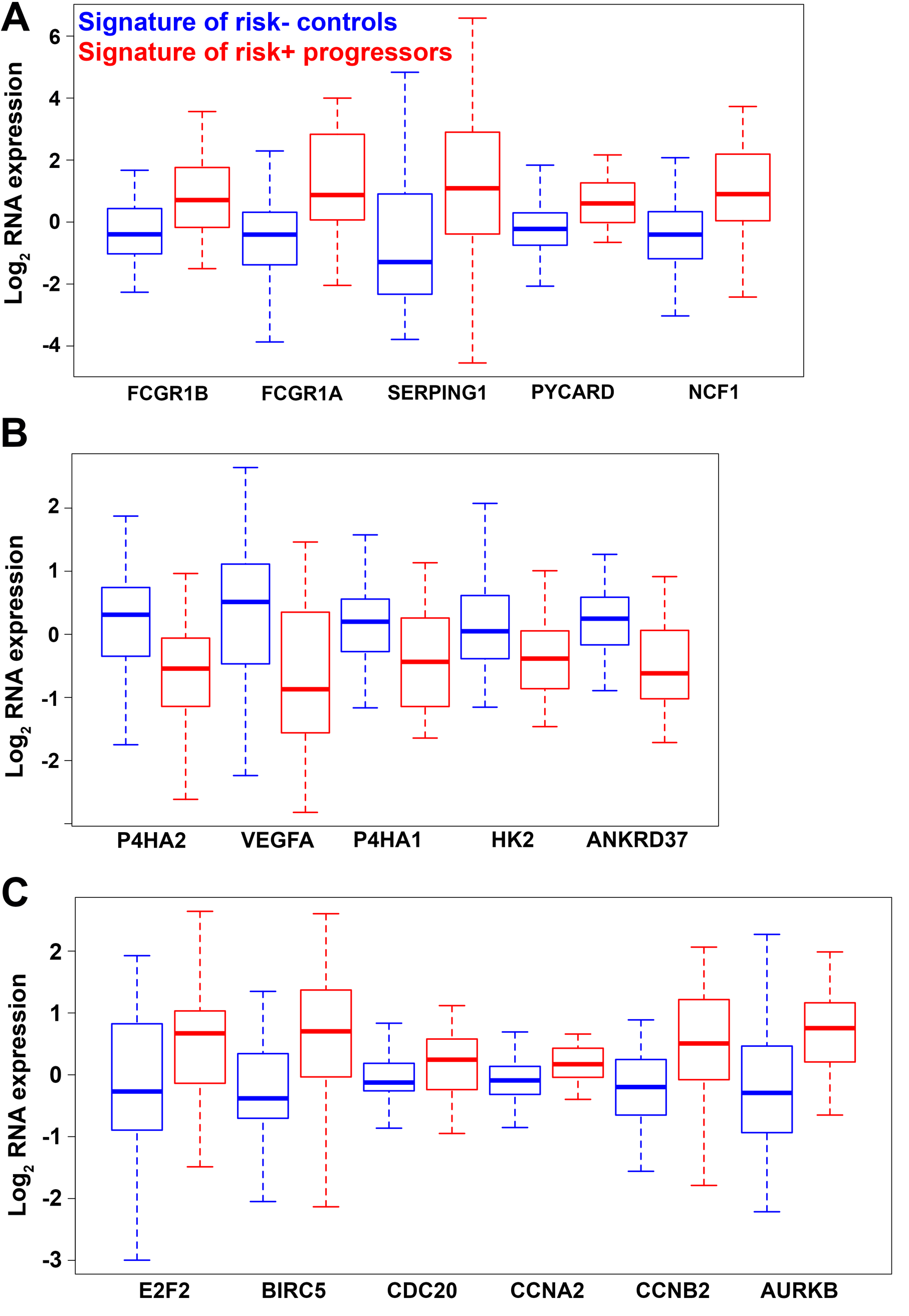

Supplement: S2 Fig — (A) Levels of selected mRNA transcripts of genes found to be differentially expressed in sorted monocytes from progressors with positive ACS signature of risk of TB, indicating an IFN response, and controls with negative ACS signature of risk of TB. Data from 31 progressors (122 progressor samples were signature-positive, 44 were negative) and 90 controls (236 control samples were signature-negative, 28 were positive) were included in the analysis and time to TB was not considered. A total of 89 genes were differentially expressed; the full set is in S8 Table. The gene modules enriched in genes differentially expressed between progressors with positive ACS signature of risk of TB and controls with negative ACS signature of risk of TB are listed in S8 Table. (B, C) Differentially expressed mRNA transcripts in sorted T cells from progressors with positive ACS signature of risk of TB, indicating IFN responses, and controls with negative ACS signature of risk of TB. Data from 31 progressors (138 progressor samples were signature-positive, 67 were negative) and 90 controls (299 control samples were signature-negative, 40 were positive) were included in the analysis and time to TB was not considered. Representative genes significantly enriched in the hypoxia (B) and cell cycle (C) modules by modular analysis, at a p-value < 0.01 and an FDR <0.3, are shown. The full set of differentially expressed T cell genes is in S6 Table and gene modules enriched in genes differentially expressed between progressors with positive ACS signature of risk of TB and controls with negative ACS signature of risk of TB are listed in S7 Table. (TIF) [file ppat.1006687.s002.tif]

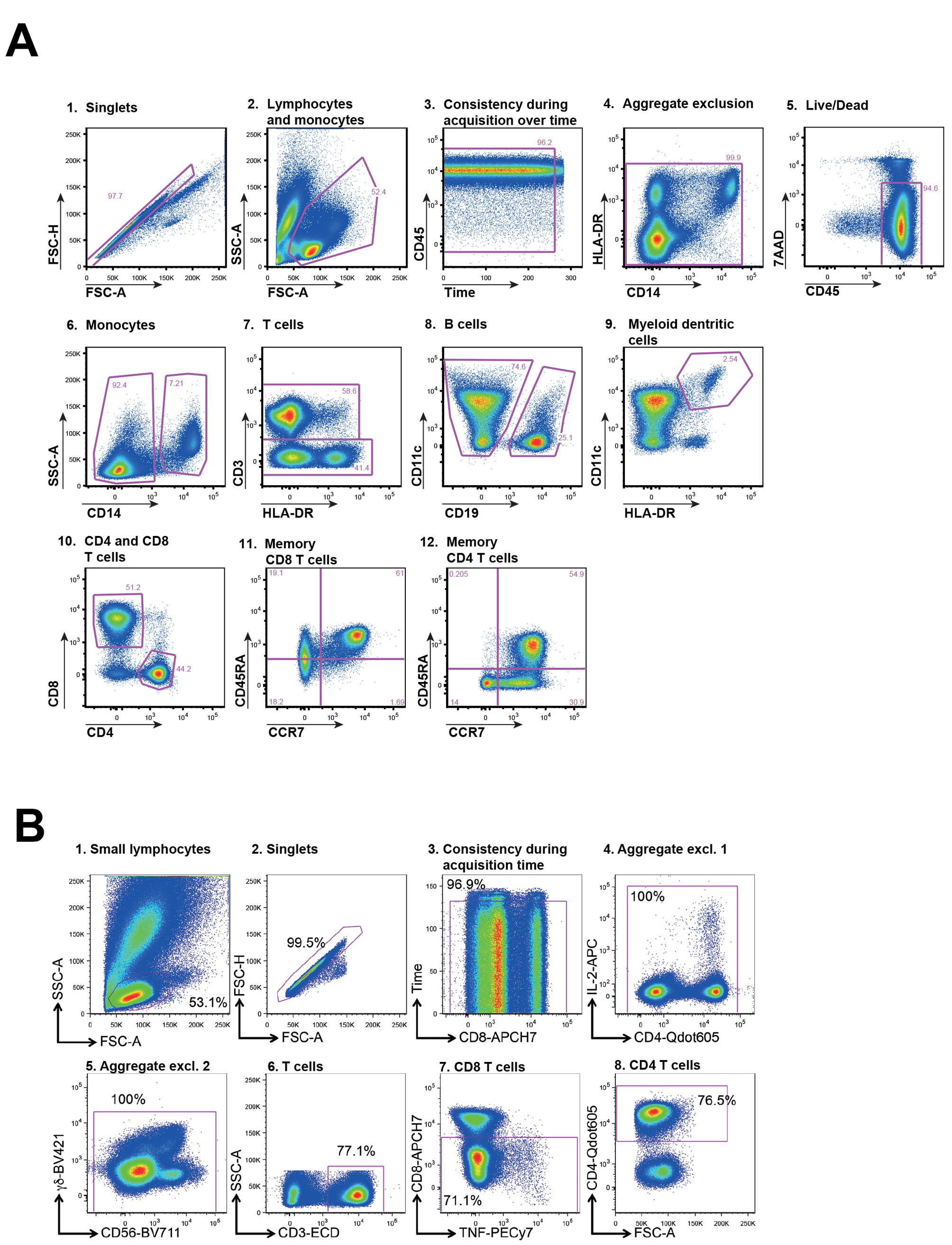

Supplement: S3 Fig — (A) Gating strategy used to measure proportions of myeloid and lymphoid cell populations in PBMC from adolescent progressors and controls. The figure shows a representative sample from an adolescent. The numbering above each plot indicates the order of gating. (B) Gating strategy used to measure frequencies of BCG-specific CD4 T cells expressing cytokines by intracellular cytokine staining assays from cryopreserved, stimulated whole blood. The figure shows a representative sample collected 3 weeks after BCG revaccination from a single donor. The sequence for gating is indicated by the numbering above the plots. Numbers within each plot indicates the proportion of cells falling into the relevant gates. (TIF) [file ppat.1006687.s003.tif]

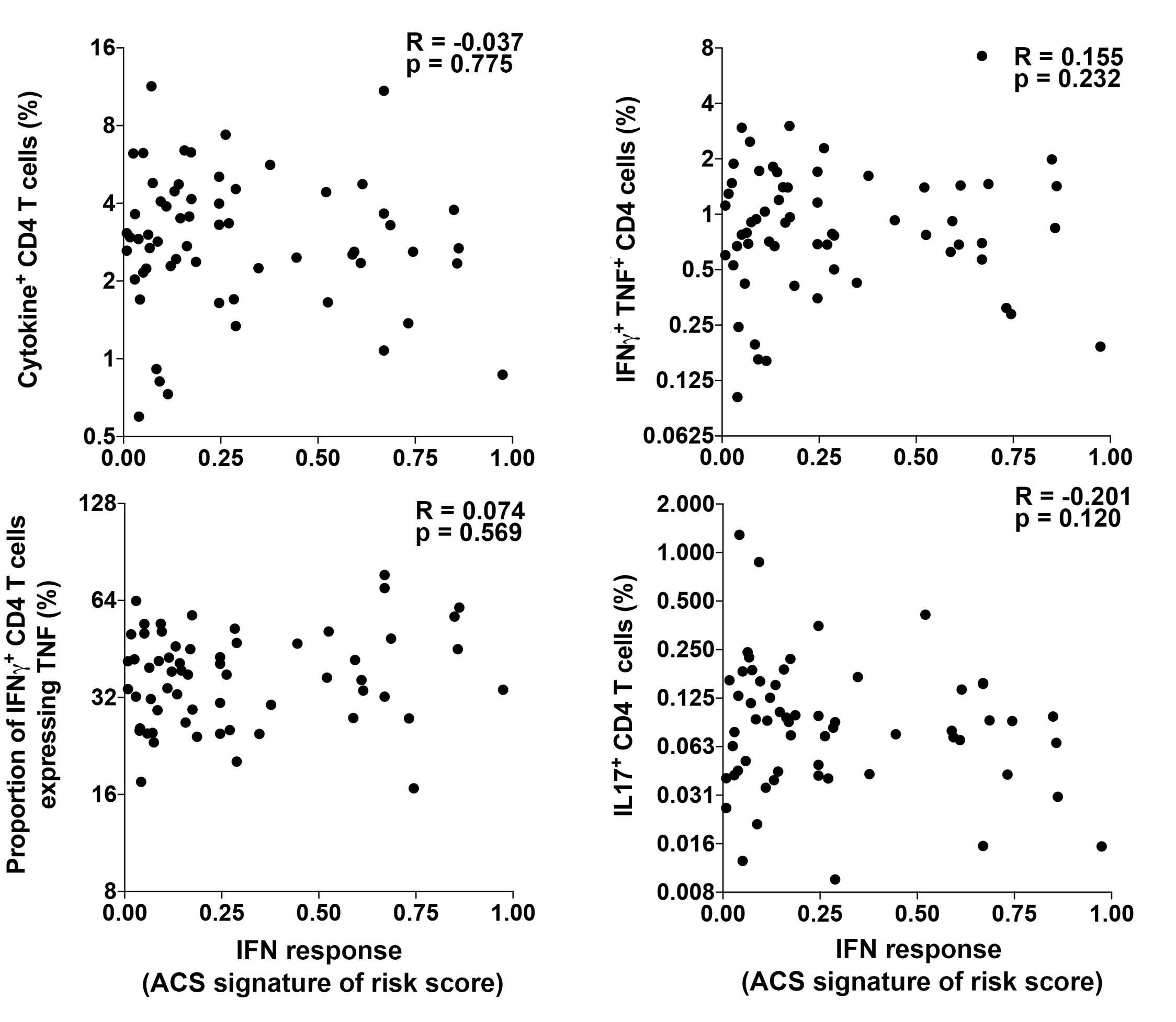

Supplement: S4 Fig — Type I/II IFN response was measured by the ACS signature of risk of TB. Shown are frequencies of total cytokine+ BCG-specific CD4 T cells, cells co-expressing IFNγ and TNF, relative proportions of BCG-specific IFNγ+ CD4 T cells co-expressing TNF and frequencies of total IL-17+ CD4 T cells. Spearman R and p-values are shown in each plot. (TIF) [file ppat.1006687.s004.tif]
