# Supplementary material for: Sequential inflammatory processes define human progression from M. tuberculosis infection to tuberculosis disease
Source: PLoS Pathog. 2017 Nov 16;13(11):e1006687. doi: 10.1371/journal.ppat.1006687 (PMC5689825; doi:10.1371/journal.ppat.1006687)
Supplement: S1 Methods — (DOCX) [file ppat.1006687.s014.docx]

**Supplementary Methods**

**Adolescent Cohort Study**

Briefly, 6,363 healthy adolescents, aged 12-18 years, were enrolled between July 2005 and April 2007 and follow-up was completed by February 2009. Approximately half of the adolescents were evaluated at enrollment and every 6 months during 2 years follow-up; the other half was evaluated at baseline and at 2 years. At enrollment and at each visit, clinical data were collected, 2.5mL blood was collected directly into PAXgene blood RNA tubes (PreAnalytiX) and blood was collected in Cell Preparation Tubes (BD Biosciences) and peripheral blood mononuclear cells and plasma were isolated using density gradient centrifugation.

Only adolescents with *M.tb* infection at enrollment, or those who developed active TB disease more than 6 months after *M.tb* infection was first detected were included in our analyses, diagnosed by a positive QuantiFERON TB GOLD In-Tube Assay (QFT, Qiagen; >0·35 IU/mL) and/or a positive tuberculin skin test (TST, 0·1mL dose of Purified Protein Derivative RT-23, 2-TU, Staten Serum Institute; >10mm). According to South African policy, QFT and/or TST positive adolescents were not given therapy to prevent tuberculosis disease. Progressors were adolescents who developed active TB disease during follow-up, defined as intrathoracic disease, with either two sputum smears positive for acid-fast bacilli or one positive sputum culture confirmed as *M.tb* complex (mycobacterial growth indicator tube, BD BioSciences). For each progressor, two matched controls who remained healthy during follow-up were selected and matched by age at enrolment, gender, ethnicity, school of attendance, and presence or absence of prior episodes of tuberculosis disease (**Table 1 and Figure 1**). Participants were excluded if they developed tuberculosis disease within 6 months of enrollment or QFT and/or TST conversion, to exclude early asymptomatic disease that could have been present at the time of evaluation, or if they were HIV infected.

The study protocols were approved by the Human Research Ethics Committee of the Faculty of Health Sciences, University of Cape Town. Written informed consent was obtained from parents or legal guardians, and written informed assent from each adolescent. Participants with diagnosed or suspected tuberculosis disease were referred to a study-independent public health physician for treatment according to national tuberculosis control programs of the country involved.

**Adult trial of BCG revaccination**

Briefly, we recruited healthy 18 to 40 year old South African adults, who were strongly TST positive (≥ 15mm induration when tested with PPD RT-23); HIV-seronegative; received BCG at birth and had a visible BCG scar. In this phase I trial, participants, recruited from the population of Worcester in the Western Cape, South Africa were randomized in parallel into two groups in a 1:1 ratio as previously described [1,2]. Participants in the first group were observed for 7 months, then vaccinated with BCG, and subsequently treated with INH 6 months later (Observation-BCG-INH: OBI group). Participants in the second group received a course of 6 months of INH within a maximum period of 7 months, followed by BCG vaccination (INH-BCG-Observation: IBO group). Danish strain 1331 BCG Vaccine SSI (Statens Serum Institut, Copenhagen, Denmark), the BCG vaccine used in the South African national immunization program and one of the most widely administered BCG vaccines worldwide, was administered intradermally at an adult dose of 2 to 8 x 10^5^ CFUs. INH (isoniazid, Westward Pharmaceutical Corporation, Eatontown, NJ, USA) was administered daily at 5mg/kg rounded up to the nearest 100mg (maximum dose 300 mg/day), and INH adherence was monitored by pill counts at clinic visits and random urine INH metabolite testing (18). All participants provided written, informed consent. The protocol was approved by the Medicines Control Council (MCC) of South Africa, Human Research Ethics Committee (HREC) of the University of Cape Town and the University Hospitals Case Medical Center institutional review board. The trial was registered on ClinicalTrials.gov (NCT01119521). Whole blood was collected in PAXGene tubes and in Sodium-Heparin tubes from participants and processed within 45 minutes of phlebotomy, as previously described (19), at enrollment, 1 month after IPT initiation, at BCG vaccination, at 3 and 5 weeks, and 1 year post-vaccination. RNA was isolated from PAXGene tubes as described above. Heparinized blood was stimulated and processed for measurement of T cell responses by whole blood intracellular cytokine staining (WB-ICS) assay, as previously described [2]. The signature of risk of TB was measured in samples collected before BCG revaccination and the functionality of the T cell response to BCG revaccination was measured 3 weeks after vaccination.

**RNA sequencing and analyses**

RNA was extracted from PAXgene tubes, globin transcripts were depleted and (GlobinClear, Life Technologies) cDNA libraries were prepared using Illumina mRNA-Seq Sample Prep Kit. RNA-Seq was performed by Expression Analysis Inc., at 30 million 50bp paired-end reads, on Illumina HiSeq-2000 sequencers. Read pairs were aligned to the hg19 human genome using gsnap, which generated a table of gene expression abundances for each sample.

Whole blood, monocyte and CD4 T cell RNA-Seq data was aligned to the hg19 human genome using gsnap [3] as in the original study [4]. Normalized gene-level expression estimates were derived from mapped read pairs following the procedure implemented previously [5]. Briefly, mapped read pairs were assigned to genes by collapsing all transcripts into a single gene model and counting the number of reads that fully overlap the resulting exons using htseq (v. 0.6.0) [6], with strict intersection and including strand information. Gene models for protein-coding genes were downloaded from Ensembl (GRCh37.74). Reads that mapped to multiple locations were only counted once and those mapping to ambiguous regions were excluded. Log_2_-transformed values of counts normalized by adjusted library counts were computed using the cpm function of the edgeR package [7]. For monocyte and CD4 T cell transcriptomic analyses, both RNASeq and qRT-PCR measurements of the ACS signature of risk of TB score (**Table S1**) were used to classify samples as positive (> 0.6 in both RNASeq and qRT-PCR) or negative (< 0.4 in both RNASeq and qRT-PCR), to ensure robust classification.

**Flow cytometry**

For the progressor and controls analyses, PBMC were stained anti-CCR7-PE (150503) at 37°C for 20 min and thereafter at 4°C for 30 mins with a cocktail of the following antibodies: CD45-FITC (2D1), CD11c-PerCP-Cy5.5 (Bu15), CD19-PE-Cy7 (SJ25C1), CD45RA-APC (550855), HLA-DR-AF700 (L243, BioLegend), CD14-Qdot585/605 (TuK4), CD3-V450 (UCHT1), CD8-Qdor655 (3B5, Invitrogen), CD4-V500 (RPA-T4) and the live/dead dye, 7AAD (ViaProbe). All antibodies and dyes were from BD Biosciences, unless otherwise indicated. Cells were acquired on a BD LSRFortessa flow cytometry and analysed using FlowJo (v9.2). Dead cells and doublets were excluded before cell subset proportions were computed. The gating strategy on a representative donor sample is shown in **Figure S1A.**

For the BCG revaccination analyses, cryopreserved, fixed whole blood samples were thawed and stained at 4°C in BD perm/wash buffer (BD) with CD3-ECD (UCHT1, Beckman-Coulter), CD4-Qdot605 (S3.5, Invitrogen), CD8-APC-H7 (SK1), TCR-γδ-BV421 (B1), CD56-BV711 (HCD56, Biolegend), IFNγ-Alexa700 (B27), TNFα-PECy7 (MAb11, eBioscience), IL-2-APC (MQ1-17H2), IL-17-Alexa488 (N49-653), IL-22-PE (IC7821P, R&D Systems). All antibodies and dyes were from BD Biosciences, unless otherwise indicated. Cells were acquired on a BD LSRFortessa flow cytometry and analysed using FlowJo (v9.2). The gating strategy on a representative donor sample is shown in **Figure S1B.**

**Transcriptomic analyses of sorted T cells and monocytes**

Cyropreserved PBMC from progressors and controls were thawed and used for *M.tb* antigen stimulation experiments to sort monocytes and T cells for transcriptomic analyses. Specifically, CD14+ monocytes were sorted by positive selection using Miltenyi CD14 microbeads on an AutoMACS Pro to a purity of >90% (verified by flow cytometry). Two x 10^5^ sorted monocytes were subsequently stimulated with 2x10^6^ CFU/ml live H37Rv *M.tb* in 0.5mL final volume, or left unstimulated for 6 h at 37°C.

Similarly, for analyses of T cells, thawed PBMC were rested at 37°C for 4-6 hours, and 1x10^6^ live PBMC were stimulated with 1x10^6^ CFU/ml live H37Rv in 0.5 mL final volume, at 37°C for 12 hr, or with pools of 15mer peptides overlapping by 10 amino acids (1μg/ml/peptide), of ESAT-6 and CFP-10, or Ag85A/Ag85B. Stimulation in with 0.27% DMSO served as the negative control. Anti-CD28 and anti-CD49d co-stimulatory antibodies (1μg/ml, BD Biosciences) were added to the peptide pool stimulated and negative control conditions. After stimulation at 37°C for 12 hours, T cells were purified from PBMC by negative selection using Miltenyi Pan-T cell isolation kit on an AutoMACS Pro (for peptide and negative controls), or manually using MACS columns under BSL-3 conditions for *M.tb*-stimulated samples to a purity of >99% (verified by flow cytometry). Purified T cells were lysed in RNeasy RLT buffer (QIAgen) while purified monocytes and *M.tb*-stimulated T cells were lysed in PrimeStore MTM buffer (Longhorn Vaccines and Diagnostics). RNA was extracted from sorted cell subsets using RNeasy Plus Micro kit (QIAgen) and subjected to RNA sequencing as described above.

**Figure S1. Selected genes and proteins found to be significantly abundant between progressors and controls.** (A) Kinetics of mRNA expression over time, expressed as log_2_ fold change between bin-matched progressors and controls and modeled as non-linear splines (dotted lines) for two representative interferon response genes and two representative inflammation genes. Light green shading represents 99% CI and dark green shading 95% CI for the temporal trends, computed by performing 2000 spline fitting iterations after bootstrap resampling from the full dataset. The relative difference in magnitude for each gene, representing the log_2_ fold change at TB diagnosis, is shown in green text. The deviation time, calculated as the time point at which the 99% CI deviates from a log_2_ fold change of 0, is indicated in red text. (B) Kinetics of plasma protein abundance over time, expressed as log_2_ fold change between bin-matched progressors and controls and modeled as non-linear splines (dotted lines) for proteins representative of the major response pathways during progression. Light purple shading represents 99% CI and dark purple shading 95% CI for the temporal trends, computed by performing 2000 spline fitting iterations after bootstrap resampling from the full dataset. The relative difference in magnitude for each protein, representing the log_2_ fold change at TB diagnosis, is shown in purple text. The deviation time, calculated as the time point at which the 99% CI deviates from a log_2_ fold change of 0, is indicated in red text.

**Figure S2. Transcriptomic signatures in monocytes and T cells associated with whole blood IFN responses.** (**A**) Levels of selected mRNA transcripts of genes found to be differentially expressed in sorted monocytes from progressors with positive ACS signature of risk of TB, indicating an IFN response, and controls with negative ACS signature of risk of TB. A total of 89 genes were differentially expressed; the full set is in **Table S8**. The gene modules enriched in genes differentially expressed between progressors with positive ACS signature of risk of TB and controls with negative ACS signature of risk of TB are listed in **Table S8**. (**B, C**) Differentially expressed mRNA transcripts in sorted T cells from progressors with positive ACS signature of risk of TB, indicating IFN responses, and controls with negative ACS signature of risk of TB. Representative genes significantly enriched in the hypoxia (**B**) and cell cycle (**C**) modules by modular analysis, at a p-value < 0.01 and an FDR <0.3, are shown. The full set of differentially expressed T cell genes is in **Table S6** and gene modules enriched in genes differentially expressed between progressors with positive ACS signature of risk of TB and controls with negative ACS signature of risk of TB are listed in **Table S7**.

**Figure S3. Flow cytometry gating strategies.** (A) Gating strategy used to measure proportions of myeloid and lymphoid cell populations in PBMC from adolescent progressors and controls. The figure shows a representative sample from an adolescent. The numbering above each plot indicates the order of gating. (B) Gating strategy used to measure frequencies of BCG-specific CD4 T cells expressing cytokines by intracellular cytokine staining assays from cryopreserved, stimulated whole blood. The figure shows a representative sample collected 3 weeks after BCG revaccination from a single donor. The sequence for gating is indicated by the numbering above the plots. Numbers within each plot indicates the proportion of cells falling into the relevant gates.

**Figure S4.** Associations between cytokine expressing CD4 T cells after stimulation of whole blood with BCG or medium (unstimulated) and the ACS signature of risk of TB (COR score), in adults from the BCG revaccination study. Type I/II IFN response was measured by the ACS signature of risk of TB. Shown are frequencies of total cytokine^+^ BCG-specific CD4 T cells, cells co-expressing IFNγ and TNF, relative proportions of BCG-specific IFNγ^+^ CD4 T cells co-expressing TNF and frequencies of total IL-17^+^ CD4 T cells are shown. Spearman R and p-values are shown in each plot.

**Supplementary References**

1. Hatherill M, Geldenhuys H, Pienaar B, Suliman S, Chheng P, Debanne SM, et al. Safety and reactogenicity of BCG revaccination with isoniazid pretreatment in TST positive adults. Vaccine. 2014;32: 3982–3988. doi:10.1016/j.vaccine.2014.04.084

2. Suliman S, Geldenhuys H, Johnson JL, Hughes JE, Smit E, Murphy M, et al. Bacillus Calmette-Guérin (BCG) Revaccination of Adults with Latent Mycobacterium tuberculosis Infection Induces Long-Lived BCG-Reactive NK Cell Responses. 2016;197: 1100–1110. doi:10.4049/jimmunol.1501996

3. Wu TD, Nacu S. Fast and SNP-tolerant detection of complex variants and splicing in short reads. Bioinformatics. 2010;26: 873–881. doi:10.1093/bioinformatics/btq057

4. Zak DE, Penn-Nicholson A, Scriba TJ, Thompson E, Suliman S, Amon LM, et al. A blood RNA signature for tuberculosis disease risk: a prospective cohort study. The Lancet. 2016;387: 2312–2322. doi:10.1016/S0140-6736(15)01316-1

5. Hoft DF, Blazevic A, Selimovic A, Turan A, Tennant J, Abate G, et al. Safety and Immunogenicity of the Recombinant BCG Vaccine AERAS-422 in Healthy BCG-naïve Adults: A Randomized, Active-controlled, First-in-human Phase 1 Trial. EBioMedicine. 2016;7: 278–286. doi:10.1016/j.ebiom.2016.04.010

6. Anders S, Pyl PT, Huber W. HTSeq--a Python framework to work with high-throughput sequencing data. Bioinformatics. 2015;31: 166–169. doi:10.1093/bioinformatics/btu638

7. McCarthy DJ, Chen Y, Smyth GK. Differential expression analysis of multifactor RNA-Seq experiments with respect to biological variation. Nucleic Acids Res. 2012;40: 4288–4297. doi:10.1093/nar/gks042
